# Microporous Polymer-Modified Glassy Carbon Electrodes for the Electrochemical Detection of Metronidazole: Experimental and Theoretical Insights

**DOI:** 10.3390/nano14020180

**Published:** 2024-01-12

**Authors:** Héctor Quiroz-Arturo, Carlos Reinoso, Ullrich Scherf, Alex Palma-Cando

**Affiliations:** 1 Grupo de Investigación Aplicada en Materiales y Procesos (GIAMP), School of Chemical Sciences and Engineering, Yachay Tech University, Hda. San José s/n y Proyecto Yachay, Urcuqui 100115, Ecuador; 2 School of Physical Sciences and Nanotechnology, Yachay Tech University, Hda. San José s/n y Proyecto Yachay, Urcuqui 100115, Ecuador; 3 Department of Chemistry, Macromolecular Chemistry and Wuppertal Center for Smart Materials @ Systems (CM@S), Bergische Universität Wuppertal, Gaußstr. 20, 42119 Wuppertal, Germany

**Keywords:** microporous polymer films, carbazole derivatives, modified glassy carbon electrodes, metronidazole detection, XPS, computational modeling

## Abstract

The persistence and potential toxicity of emergent pollutants pose significant threats to biodiversity and human health, emphasizing the need for sensors capable of detecting these pollutants at extremely low concentrations before treatment. This study focuses on the development of glassy carbon electrodes (GCEs) modified by films of poly-tris(4-(4-(carbazol-9-yl)phenyl)silanol (PTPTCzSiOH), poly-4,4′-Di(carbazol-9-yl)-1,1′-biphenyl (PCBP), and poly-1,3,5-tri(carbazol-9-yl)benzene (PTCB) for the detection of metronidazole (MNZ) in aqueous media. The films were characterized using electrochemical, microscopy, and spectroscopy techniques, including scanning electron microscopy (SEM) and X-ray photoelectron spectroscopy (XPS). Monomers were electropolymerized through cyclic voltammetry and chronoamperometry techniques. Computational methods at the B3LYP/def2-TZVP level were employed to investigate the structural and electrochemical properties of the monomers. The electrochemical detection of MNZ utilized the linear sweep voltammetry technique. Surface characterization through SEM and XPS confirmed the proper electrodeposition of polymer films. Notably, MPN-GCEs exhibited higher detection signals compared to bare GCEs up to 3.6 times in the case of PTPTCzSiOH-GCEs. This theoretical study provides insights into the structural, chemical, and electronic properties of the polymers. The findings suggest that polymer-modified GCEs hold promise as candidates for the development of electrochemical sensors.

## 1. Introduction

Since the pioneering work of Heeger, MacDiarmid, and Shirakawa almost 50 years ago, interest in the field of conducting polymers (CPs) has significantly increased [1]. In recent years, several studies have been carried out on CP applications in solar cells [2], batteries [3], supercapacitors [4], light-emitting diodes [5], transistors [6], electrochromic devices [7,8], actuators [9], and chemical and electrochemical sensors for drug, environmental pollution, and food analysis as well as for medical diagnosis [10,11,12,13]. The presence of emergent pollutants in water bodies has a negative impact on ecological systems and living organisms [14,15]. A distinctive group of these contaminants are pharmaceutical emergent pollutants, which have adverse human health effects even at very low concentrations. The concentration of these pollutants has increased sharply due to the high demand for pharmaceuticals and personal care products as well as their structural stability, which extends their period of degradability and persistence in the environment [16]. Despite its medicinal properties, metronidazole (MNZ) contributes to increases in these problems. It is a soluble and non-biodegradable compound that bioaccumulates in aquatic ecosystems [17]. Furthermore, evidence suggests that MNZ has mutagenic, genotoxic, and carcinogenic side effects [18]. Therefore, action is urgently required for the detection of MNZ. So far, several techniques have been reported for detecting MNZ, among which electrochemical methods employing modified electrodes are the most prominent [18,19]. Electrochemical sensing of MNZ is based on the electrochemical reduction of the nitro group (-NO_2_) into the hydroxylamine group (-NHOH), a process involving four protons and four electrons [20,21]. Sensors based on glassy carbon electrodes modified by carbon nanotubes [22], silver nanoparticles/sulfonate functionalized graphene [23], MoS_2_/graphitic carbon nitride composites [24], and poly(alizarin red s) [25] and a wide variety of chemically modified electrodes have been developed to overcome the poor sensitivity and selectivity of bare electrodes [26]. A glaring example of this is the electrochemical sensor based on graphene-like carbon architecture and a polythionine-modified glassy carbon electrode that exhibits high sensitivity and good selectivity and improves the detection signal by 144% compared with electrochemically treated GCEs [18].

**Figure 1:**
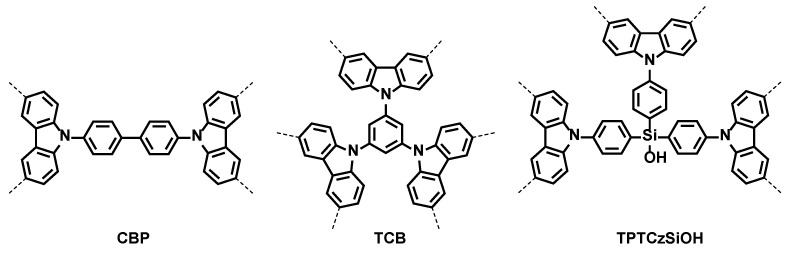
Chemical structure of multifunctional carbazole-based monomers: CBP, TCB, and TPTCzSiOH. The dashed bonds indicate the oxidative linking sites available on each molecule.

In the field of CPs, polycarbazoles have gained increasing research attention due to their application in blue-emitting OLEDs and solar cells [27]. Microporous polymer networks (MPNs) based on multifunctional carbazole derivatives possess excellent electrical and photoelectric properties, and the rigid conjugated structure provides them with high specific surface areas and chemical stability [28,29]. Porous polycarbazoles may be prepared by oxidative coupling, the Friedel–Crafts reaction, nitrile-based trimerization, and C–C coupling reactions [28]. In addition to these chemical methods, electrochemical polymerization (EP) has also been employed for the preparation of porous polycarbazole films [30,31]. However, the electropolymerization of carbazole requires a high potential for oxidation, and the polymerization rate is slow. For this reason, carbazole has been subjected to modifications that lower the oxidation potential of the monomer and allow it to control the optical and electronic properties of the resulting carbazole-based polymer [32]. We have previously reported the electrochemical generation and characterization of MPN films, starting from 4,4′-di(carbazol-9-yl)-1,1′-biphenyl (CBP), 1,3,5-tri(carbazol-9-yl)benzene (TCB), and tris(4-(carbazol-9-yl)phenyl)silanol (TPTCzSiOH) monomers (see Figure 1) with two and three carbazole substituents [33,34]. These MPN films showed high specific surface areas of 104 m^2^g^−1^, 748 m^2^g^−1^, and 165 m^2^g^−1^ for poly-4,4′-di(carbazol-9-yl)-1,1′-biphenyl (PCBP), poly-1,3,5-tri(carbazol-9-yl)benzene (PTCB), and poly-tris(4-(carbazol-9-yl)phenyl)silanol (PTPTCzSiOH), respectively, by direct measure with Kr sorption isotherms. Potential applications for MPN-modified electrodes were tested for the electrochemical detection of prototypical nitroaromatic analytes such as nitrobenzene, 1,3,5-trinitrobenzene, and 2,4,6-trinitrophenol. 

The development of electrochemical sensors based on microporous carbazyl polymer films is a promising candidate for the detection of MNZ. To the best of our knowledge, this is the first report where CBP, TCB, and TPTCzSiOH monomers are electropolymerized and characterized on GCEs for metronidazole detection. The microporosity of these polymers combined with their electron-rich nature provides a potential opportunity for boosting the current response in electrochemical detectors. Furthermore, the presence of the hydroxyl group in TPTCzSiOH is expected to enhance the hydrophilicity of the MPN film, thereby increasing its compatibility with aqueous media. In this context, the evaluation of the structural and electrochemical stability, reversibility, and adherence properties is studied for PCBP, PTCB, and PTPTCzSiOH films on GCEs as well as the surface chemical composition and morphology. Moreover, the feasibility of modifying GCEs for MNZ detection and an analysis of the monomer structures using a computational approach to understand and predict the properties of the conducting polymers are reported within this work.

## 2. Materials and Methods

### 2.1. Reagents and Equipment

All reagents used during this research project were purchased from commercial sources, except for TPTCzSiOH, which was previously synthesized as reported in [34], and MNZ was purified from commercial tablets ($C_{6}H_{9}N_{3}O_{3}$, 500 mg, La Santé). Commercial products were used as received without further purification: acetonitrile (HPLC reagent, ≥99.95%, Fisher Chemical, Waltham, MA, USA), dichloromethane (HPLC reagent, ≥99.9%, Fisher Chemical), tetrabutylamonium perchlorate (for electrochemical analysis, ≥99.0%, Sigma-Aldrich, St. Louis, MI, USA), ethanol (70%, Distribuidora M&M-Ibarra, Ibarra, Ecuador), 4,4′-Di(carbazol-9-yl)-1,1′-biphenyl (CBP, 97%, Sigma-Aldrich), 1,3,5-tri(carbazol-9-yl)benzene (TCB, 97%, Sigma-Aldrich), potassium chloride (P.A), potassium phosphate dibasic (99.8%, Fisher Chemical), potassium phosphate monobasic (99.6%, Fisher Chemical), and methanol (99.8%, Fisher Chemical). Autolab PGSTAT128N (Metrohm, Barendrecht, The Netherlands) and Dropsense µStat 300 (Metrohm) were used for the electropolymerization of CBP, TCB, and TPTCzSiOH monomers; the electrochemical characterization of their respective polymer films; and the electrochemical detection of MNZ. SEM micrographs and EDS spectra were measured with Phenom ProX (Thermo Fisher Scientific, Waltham, MA, USA). X-ray photoelectron spectroscopy was performed using PHI VersaProbe III (Physical Electronics, Chanhassen, MN, USA) equipped with a 180 hemispherical electron energy analyzer.

### 2.2. General Procedure for Electrochemical Polymerization and Characterization

Electrochemical polymerization was performed in a three-electrode configuration cell containing acetonitrile (ACN)/dichloromethane (DCM) (1:4) as the solvent mixture and tetrabutylammonium perchlorate (TBAP) as the supporting electrolyte (0.1 M), with a monomer concentration of 0.1 mM. For electrochemical characterization, a monomer-free solution of 0.1 M TBAP in ACN was employed. The cell was attached to a potentiostat/galvanostat and a thermostat at 25 °C. Glassy carbon (for monomer and polymer characterization in addition to electrochemical sensing of MNZ) and indium tin oxide (ITO, for surface characterization) were used as working electrodes (WEs) in combination with a platinum wire/sheet counter electrode (CE) and Ag^0^/${AgNO}_{3}$ (0.1 M ${AgNO}_{3}$, 0.1 M TBAP, 0.60 V vs. SHE, nonaqueous reference) or Ag^0^/AgCl (3 M KCl, 0.21 V vs. SHE, aqueous reference) as reference electrodes (REs). Potentiodynamic or potentiostatic regimes were applied for the generation of microporous films on the electrodes. Prior to use, WEs were treated as follows: the GCE surface was polished to a mirror with 0.05 μm alumina powder; then, the electrode was rinsed thoroughly with deionized water to remove any adsorbed material. The ITO electrode was sonicated in three different media: first in soapy water, then in 1:1 deionized water and ethanol, and, finally, in DI water. Both electrodes were dried using an air stream at room temperature.

#### 2.2.1. Determination of the Optimal Potential Range for Electropolymerization

CBP, TCB, and TPTCzSiOH monomers were electropolymerized on GCEs via cyclic voltammetry from 0 V to different values of the anodic switching potential (E_+λ_ between 0.8 V and 2.0 V) during 3 cycles at a sweeping rate of 0.1 V/s. Electropolymerization was carried out in 5 mL ACN/DCM (1:4) as the solvent mixture and 0.1 M TBAP as the supporting electrolyte, with a monomer concentration of 0.1 mM. A platinum wire CE and Ag/${AgNO}_{3}$ nonaqueous reference were employed. Before each electropolymerization process, the GCE was properly cleaned and treated as mentioned above. 

#### 2.2.2. Electrochemical Stability, Reversibility, and Adherence of Deposited Films

Monomers at a concentration of 0.1 mM were electropolymerized using a CV technique from 0 V to an oxidative switching potential value of 1.1 V during 20 cycles. After EP and washing of the deposits with ACN and DCM, the polymer-modified GC electrodes were placed in 5 mL of an ACN solution containing 0.1 M TBAP at 25 °C. Then, 30 voltammetric cycles (0–1.0 V) of each polymer film were registered. The sweeping rate employed for both sections was 0.1 V/s. Immediately after this, two voltammetric cycles of each deposit were recorded from 0 to 1.0 V at different sweeping rates (0.2, 0.15, 0.1, 0.05, 0.02, and 0.01 V/s) in the same monomer-free solution. A platinum wire CE and Ag/${AgNO}_{3}$ reference electrode were used in these measurements.

#### 2.2.3. Spectroscopy and Microscopy Characterization of Polymer Films

Thin polymer films were produced on the ITO surface by 13 cyclic voltammograms at 0.1 V/s from 0 to 1.1 V for TPTCzSiOH and from 0 to 1.2 V for CBP and TCB. Monomers at a concentration of 0.1 mM were electropolymerized in media containing 10 mL ACN/DCM (1:4) and 0.1 M TBAP. A platinum sheet CE and nonaqueous (Ag/${AgNO}_{3}$) reference were employed. After polymerization, the polymer-modified ITO electrode was rinsed with ACN and DCM to remove any moiety of the adsorbed monomer and TBAP. The XPS spectra were acquired employing a monochromatized Al Kα source with an energy of 1486.6 eV. Energy bandpasses of 255 kV and 55 kV were applied during the survey and high-resolution operations, respectively. The spot size diameter utilized for the measurements was 100 μm. For SEM and EDS analyses, modified ITO electrodes were placed in a charge-reduction sample holder and covered with isopropanol-based graphite paint to generate electrical contact. Finally, micrographs and spectra measurements were taken at an acceleration voltage of 15.0 kV at 1 Pa.

### 2.3. Sensing Application

#### 2.3.1. Metronidazole Purification

Commercial MNZ tablets were stripped of their outer coating and then crushed in a pestle and mortar. The resulting powder was dissolved in methanol and the non-soluble excipients were filtered out. Then, the solution was heated to 60 °C, and 1 g of activated carbon was added under constant stirring. After this, the mixture was hot-filtered to remove the activated carbon. Finally, it was cooled at room temperature until crystallization of MNZ was observed, which was then vacuum-filtered and dried. FTIR and NMR spectra are shown in Figures S1 and S2 of the Supplementary Materials.

#### 2.3.2. The Modification of Glassy Carbon Electrodes and the Electrochemical Detection of MNZ

Thin films on GC surfaces were produced by applying a stabilizing potential of 0 V for 10 s, followed by an oxidative potential of 1.1 V for 200 s, to start polymerization. The polymerization media consisted of 5 mL ACN/DCM (1:4) and 0.1 M TBAP, with a monomer concentration of 0.1 mM. A platinum wire CE and Ag/${AgNO}_{3}$ reference electrode were used. Then, the deposits were discharged by applying a potential of 0 V for 60 s. To proceed with the electrochemical detection, the modified electrodes were washed with ACN and DCM and dried at RT. Prior to the electrochemical detection of MNZ, the polymer-modified GC electrodes were placed in 5 mL of an aqueous solution containing 0.1 M potassium chloride and 0.1 M phosphate buffer (pH 7) under a nitrogen atmosphere at 25 °C. Then, these were treated by linear sweep potential from 0 to −1.0 V at a scan rate of 0.1 V/s until a stable LSV curve was obtained. A platinum wire counter electrode and an aqueous reference electrode (Ag◦/AgCl) were used for these tests. After this, MNZ stock solution was added to obtain a final concentration of 50 µM, followed by LSV measurements in the same potential range and with the same scan rate mentioned above.

### 2.4. Computational Approach

The structures of all studied molecules were built with Avogadro. A pre-optimization step was performed using the semi-empirical GFN2-xTB [35] with xtb [36] (version 6.6.0) program and the extreme optimization level. Then, those optimized structures were used as a starting point for density functional theory (DFT) calculations in the gas phase. The Becke three-parameter hybrid functional combined with the Lee, Yang, and Parr correlation functional (B3LYP) [37] was selected for calculations. The optimization was performed with the ORCA [38] (version 5.0.4) program using the def2-SVP and finally refined with the def2-TZVP basis set. The Orca inputs were generated using the Avogadro program. Dispersion correction (D3BJ) [39] and the frontier molecular orbital (FMO) analysis were incorporated in the input file of the final geometric optimization step. The band gap energy (E_g_) of the proposed molecules was calculated using Equation (1).

(1) $$E_{g}=E_{LUMO}-E_{HOMO}$$

where E_HOMO_ and E_LUMO_ are the energy of the HOMO and LUMO levels, respectively. The Multiwfn program [40,41] (version 3.8) was employed for molecular electrostatic potential map (MEP) calculations; then, MEPs were visualized using VMD 1.9.3. [42]. For this, the gbw output files from the Orca optimization process were converted to molden files using the orca 2 mkl utility as inputs for Multiwfn. 

## 3. Results and Discussion

### 3.1. Electrochemical Polymerization and Characterization

#### 3.1.1. The Determination of the Anodic Potential Range for the Electropolymerization of CBP, TCB, and TPTCzSiOH Monomers

**Figure 2:**
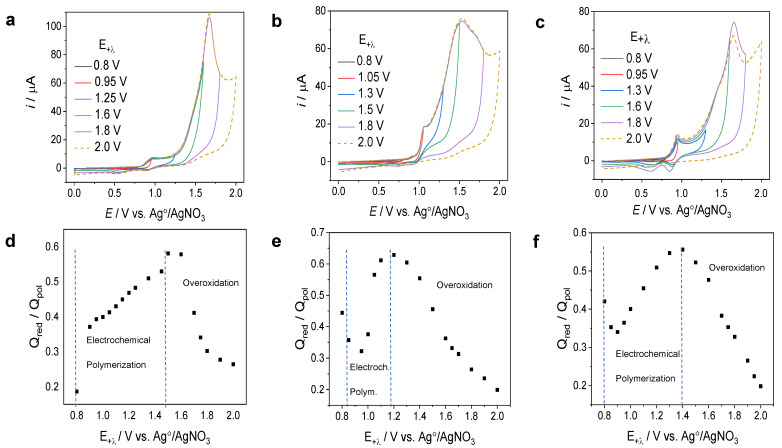
First voltammetric cycle from 0 V to different values of the anodic switching potential (E_+λ_) for the EP of (**a**) CBP, (**b**) TCB, and (**c**) TPTCzSiOH. Dependence of the ratio of reduction (Q_red_) and polymerization charge (Q_pol_) on the value of E_+λ_ applied in the first cycle for (**d**) CBP, (**e**) TCB, and (**f**) TPTCzSiOH. The sweeping rate was 0.1 V/s. Abscissa values in volts vs. Ag/AgNO_3_.

The optimal anodic potential for the electropolymerization of three monomers on GCEs was studied via cyclic voltammetry, measuring the first cycle at different switching potentials. Solutions of 0.1 mM of the monomers in acetonitrile/dichloromethane (1:4) mixtures and 0.1 M of TBAP as a supporting electrolyte were used in a three-electrode cell. A GC disk electrode was used as the working electrode (WE), Pt wire was used as the counter electrode (CE), and Ag/AgNO_3_ (0.1 M with 0.1 M TBAP) was used as the reference electrode. From the first cyclic voltammograms of each monomer (see Figure 2a–c), the polymer reduction charge (Q_red_) and polymerization charge (Q_pol_) were obtained by the integration of the area under the curve of the cathodic and anodic scans, respectively [43,44]. It was found that the anodic switching potential for all monomers could be changed within a broad range, especially for CBP. As shown in Figure 2d, there were three characteristic regions for the Q_red_/Q_pol_ vs. E plots, including the optimal region for electrochemical polymerization and overoxidation and the region of soluble oligomer formation or non-polymerization. In the case of CBP, a decrease in anodic switching potential (E_+λ_) to potentials below 0.9 V resulted in a decrease in the Q_red_/Q_pol_ ratio due to the formation of soluble oligomers. On the other hand, a value of E_+λ_ above 1.6 V gave rise to the overoxidation/degradation of the polymer film [44]. In other words, if the applied voltage was insufficient, polymerization either did not take place at all or only occurred up to the formation of low-molecular-weight oligomers. Conversely, excessive voltage led to overoxidation, causing the development of structural defects. The same trend for deviations of E_+λ_ from the optimal ranges was observed for TCB and TPTCzSiOH (see Figure 2e,f). For TPTCzSiOH polymerization, the anodic switching potential (E_+λ_) could be changed from 0.9 V to 1.5 V, while, for TCB, the optimal values for E_+λ_ were within the 0.95 V to 1.3 V range. These potential ranges for oxidative electrochemical polymerization give valuable insight into the optimal working potential to achieve more efficient polymerization without decreasing the electroactivity of the polymer film, which, in turn, might enhance the sensing properties of electrodes.

#### 3.1.2. Electrochemical Polymerization of the CBP, TCB, and TPTCzSiOH Monomers

**Figure 3:**
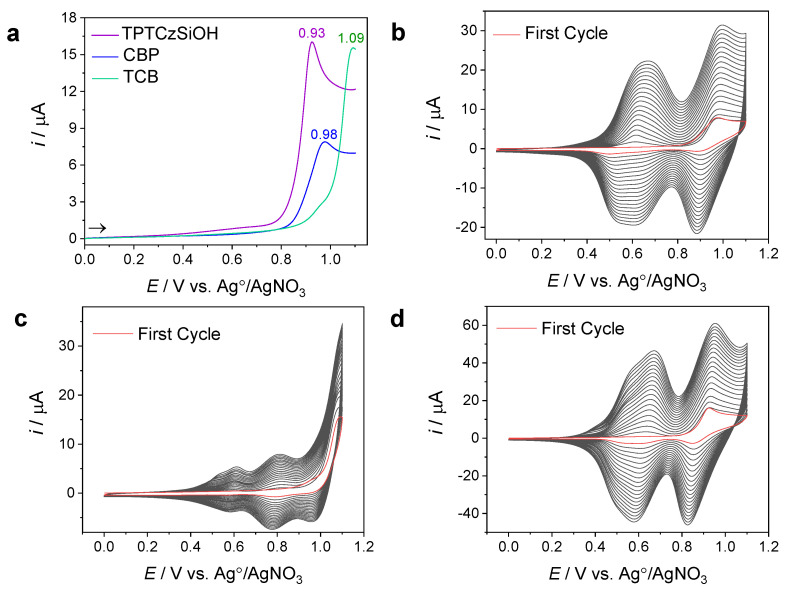
(**a**) The first anodic sweep voltammogram of each monomer and twenty cyclic voltammograms with the glassy carbon electrode carried out for 0.1 mM solutions of (**b**) CBP, (**c**) TCB, and (**d**) TPTCzSiOH monomers in acetonitrile/dichloromethane (1:4) mixtures with 0.1 M TBAP as a supporting electrolyte. Cyclic voltammograms were recorded from 0 V to 1.1 V at a scan rate of 0.1 V/s. Abscissa values in volts vs. Ag/AgNO_3_.

From the optimal potential ranges, a switching potential of 1.1 V was chosen, and all three monomers were electropolymerized by means of twenty successive CVs from 0 V vs. Ag/AgNO_3_ to 1.1 V at a sweep rate of 0.10 V/s. Figure 3a shows the first oxidation scan of CBP, TCB, and TPTCzSiOH. TPTCzSiOH oxidized onto a GCE in the ACN-DCM/TBAP system, with the first monomer oxidation peak at around 0.93V, followed by CBP at 0.98 V and TCB at 1.09 V. Similar results were reported for the electropolymerization of the three monomers with a platinum electrode [33,34]. The first oxidation peaks were attributed to the oxidation of carbazole groups in the forward scan, leading to radical cations that coupled to give rise to dimeric cations and were then reduced to 3,3-dicarbazole dimers in the reverse scan [45,46]. The high oxidation potential required for TCB in contrast to CBP and TPTCzSiOH may suggest that the electronic effect of the structural groups is rather weak [47], e.g., the structural groups of TCB have the least tendency to donate electrons [48]. The inherent steric hindrance of each monomeric structure may be another factor contributing to this effect [34]. The CBP oxidation current peak was lower than the TPTCzSiOH and TCB oxidation current peaks; this reflects the low amount of oxidizable carbazole moieties of CBP with respect to TPTCzSiOH and TCB. Similar values between the monomer oxidation peak currents of TPTCzSiOH and TCB were observed related to the same number of electroactive groups. 

During the second and subsequent cycles, reversible oxidation/reduction peaks were formed due to the charging/discharging of the polymer matrix [49]. The features of these peaks were different for each of the three polymers studied. For both PCBP and PTPTCzSiOH, there were two well-developed, reversible couples of redox peaks in the range of 0.4 V to 1.1 V (see Figure 3b,d), while, for PTCB, there were two oxidation and three reduction peaks (see Figure 3c). As the number of potential sweep cycles increased, the peak current increased gradually, thus indicating the progressive formation and growth of the polymer films on the electrode surface [33]. The increase in the current with successive cycles was small for PTCB compared to PTPTCzSiOH, reflecting a slow polymer deposition within the range of the polymer matrix response [47,50]. Similar CV curves have been observed for films synthesized potentiodynamically with platinum and ITO electrodes, suggesting a comparable redox behavior of the three molecules on GC, Pt, or ITO electrodes [33,34].

#### 3.1.3. Electrochemical Stability and Reversibility and the Adherence of the PCBP, PTCB, and PTPTCzSiOH Films on GCEs

A multicycle experiment was employed to evaluate the stability and reversibility of MPN films in a monomer-free solution. Figure S3 shows cyclic voltammograms from scan 2 (blue line) to scan 30 (red line) of each polymer film and the variation in the anodic (Q_a_) and cathodic (Q_c_) charges and the Q_c_/Q_a_ ratio. The generated PCBP film displayed the highest stability and reversibility of the three polymers (see Figure S3a). The percentage of the decrease in the cathodic current (%Q_c_ loss) gave further information about the electrochemical stability (see Table S1). PCBP showed better stability than PTCB and PTPTCzSiOH in 0.1 M TBAP ACN solutions (23.0%, 40.9%, and 36.7%, respectively). The electrochemical reversibility of this conducting polymer was confirmed by obtaining the Q_c_/Q_a_ ratio vs. the cycle graph number, which remained almost constant and close to 1 after the first ten cycles (see Figure S3d) [43]. PTPTCzSiOH showed a similar reversible profile to that of PCBP (see Figure S3c,f). In contrast, PTCB film had the lowest reversibility as the Q_c_/Q_a_ ratio began to be constant after the first twenty cycles, reaching a maximum Q_c_/Q_a_ value of 0.86 (see Figure S3b,e and Table S1), 0.14 below the ideal value for a stable and reversible system. A common feature of each polymer film was the strong current observed during the first cycle followed by a sharp drop, attributed to a change in the chemical structure of the polymer [48]. The successive decrease in the anodic and cathodic charge values was related to (i) the loss of electroactive material (e.g., short-chain oligomers) from the modified GCE surface during the expansion–contraction of the polymer matrix provoked by redox cycling [43] and (ii) the so-called “charge trapping” phenomena, in which certain counterions introduced during the oxidation process still get confined within the polymer matrix after the discharging of the film [51]. Both factors impact the faradaic current by reducing the number of available electroactive sites for charging and discharging processes during redox cycling. The characteristic features of the stability and reversibility of the polymer films described above can be seen graphically in each voltammogram. Two well-developed and reversible redox couples were observed for PTPTCzSiOH and PCBP (see Figure S3a,c). PTCB showed three redox couple responses at the beginning that became diffuse after a few cycles, especially the first couple at 0.55 V (see Figure S3b). In all cases, the redox response was affected between scans, as can be seen from the increasing distance between the second scan and subsequent cycles, thus reflecting the low reversibility and stability of the polymer films under the applied potential range. This may be associated with the presence of dissolved oxygen or trace water in the polymerization media and monomer-free solutions [48] since both experiments were carried out under non-purged conditions and the solvents were not anhydrous. It has been reported that the use of $N_{2}$ purging increases the degree of electrochemical reversibility as films show stable responses upon extended cycling, in contrast to the non-purged electrolyte [52,53,54]. 

**Figure 4:**
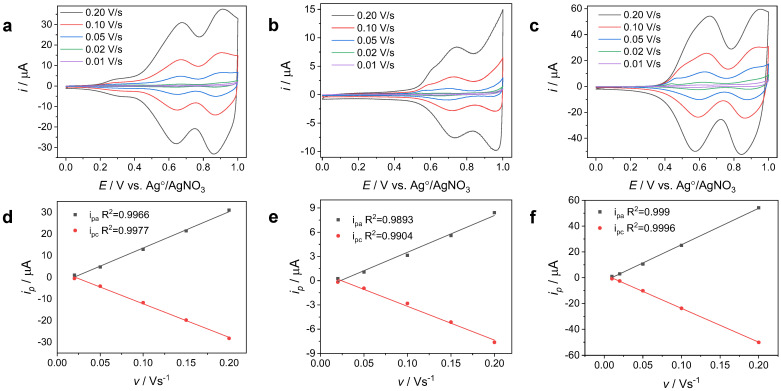
The cyclic voltammograms recorded at different scan rates from 0 V vs. Ag/AgNO_3_ to 1 V for (**a**) PCBP-, (**b**) PTCB-, and (**c**) PTPTCzSiOH-modified GCEs in a monomer-free solution. Dependence of the anodic and cathodic current peaks (i_pa_ and i_pc_) on the sweeping rate (v) for (**d**) PCBP-, (**e**) PTCB-, and (**f**) PTPTCzSiOH-modified GCEs.

Figure 4 shows the cyclic voltammograms and scan rate analyses of the polymer films. The anodic/cathodic peak currents (i_p_) followed a linear trend in a wide range of scan rate values (20 mV/s < v < 200 mV/s). This linear relationship is characteristic of a well-adhered electroactive polymer film on the electrode surface [51,55]. This means that the redox process occurring between the modified electrode and the monomer-free solution was kinetically controlled. In this sense, all three films adhered well to the electrode; the PTPTCzSiOH film had greater adhesion to the GC surface since it had the best linear fit (see Figure 4f), followed by the PCBP and PTCB deposits (see Figure 4d,e). 

### 3.2. Superficial Characterization of MPN Films

#### 3.2.1. Surface Chemical Analysis of MPN Films

**Figure 5:**
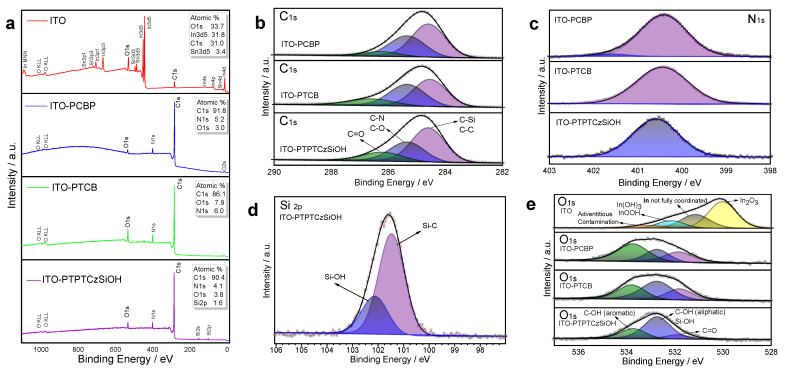
XPS spectra of the (**a**) survey scan of ITO electrode before and after electrodeposition of polymer films and high-resolution spectra of (**b**) C1s, (**c**) N1s, (**d**) Si2p, and (**e**) O1s of MPN-modified ITO electrodes.

An investigation of the surface chemical composition of the ITO glass electrodes, both before and after the electrodeposition of the polymer films, was carried out through X-ray photoelectron spectroscopy (XPS). In Figure 5a, comparative XPS survey spectra are presented for the bare ITO electrode (red line) and polymer-modified ITO electrodes: ITO/PCBP (blue line), ITO/PTPTCzSiOH (green line), and ITO/PTCB (black line). The survey spectrum of the pristine ITO electrode revealed the presence of oxygen (O), indium (In), and tin (Sn), attributed to the ITO composition, along with a carbon core level (C) associated with surface contamination (C–O–H, C–(C,H), C ꞊ O… bonds) [56,57,58,59,60]. On the other hand, the spectra of all MPN-modified electrodes indicated increased carbon and nitrogen peaks, attributable to the organic composition of the polymers. Additionally, silicon (Si) was observed solely in the electrode modified with PTPTCzSiOH, confirming the presence of its corresponding polymeric film. The atomic percentage suggests that O1s core-level photoelectrons were still present, indicating hydrocarbon contamination.

Further analysis of the high-resolution XPS spectra over C1s, O1s, N1s, and Si2p core levels was conducted to investigate the chemical bonding states. Figure 5b illustrates the deconvolution of the C1s high resolution for each polymer-modified electrode, revealing three peak components: the first at 284.6 eV, related to C-C hydrocarbon bonds; C-N and C-O at 285.3 eV, from the nitrogen-containing ring of carbazole units and adventitious hydrocarbons; and C = O at 286.4 eV, due to adventitious hydrocarbons [60,61]. In the case of ITO-PTPTCzSiOH, the full width at half maximum (FWHM) was reduced due to the contribution of the C-Si. For the N1s core level, the narrow full width at half maximum (FWHM) observed implied a singular dominant chemical environment. The N1s peak at 400.5 eV was assigned to N-C carbazole (see Figure 5c). Figure 5d shows the Si2p spectrum for the PTPTCzSiOH-modified electrode, divided into two main peaks: 101.5 eV corresponding to Si-C binding and 102.1 eV corresponding to Si-O in the silanol functional group [62,63]. The atomic percentage indicated about 74% Si-C and 26% Si-OH, aligning with the bond type distribution in the molecular structure. As depicted in Figure 5e, the deconvolution of the O1s feature unveiled distinct variations in the oxygen environments. In the case of indium tin oxide (ITO), four peaks were obtained at (i) 530.0 eV, related to metallic oxides such as In_2_O_3_ [64,65]; (ii) 531.2 eV, assigned to oxygen atoms neighboring oxygen defect sites that donate some of their electron density to not fully coordinated indium atoms [66]; (iii) 532.2 eV, related to In(OH)_3_ or InOOH on the surface; and (iv) 533.2 eV, which may be due to adventitious contaminants [67,68]. In the MPN films, the primary oxygen peak revealed only organic composition, with an absence of metal oxides (e.g., from the ITO underlayer). The oxygen signal in these polymeric materials was mainly attributed to adventitious contamination. Three peaks were obtained for all polymeric samples at (i) 531.9 eV, related to C = O; (ii) 532.8 eV, assigned to aliphatic C-OH (also Si-OH for PTPTCzSiOH); and (iii) 533.8 eV, attributed to aromatic C-OH [69]. 

The structural stability of the PTPTCzSiOH film was further investigated by applying an oxidative potential to the MPN. Electropolymerization of the TPTCzSiOH film on ITO was accomplished by cyclic voltammetry, resulting in the continuous growth of the faradaic current (see Figure S4a). Afterwards, the ITO-PTPTCzSiOH electrode was introduced in a monomer-free solution applying a fixed oxidative potential of 1 V for 30 min. At initial times, a high current response was attributed to the oxidative doping of the PTPTCzSiOH film, as seen in the chronoamperogram in Figure S4b. The current gradually decreased, stabilizing at values ca. 2 μA, which indicated the overoxidation of the film. This phenomenon might cause the loss of the charging capacity of this material (vide supra). The structural stability of the film was analyzed by XPS of the PTPTCzSiOH film after dedoping at 0 V (see Figure S5). The XPS spectroscopy analysis revealed that there were not any important chemical changes in the C1s (see Figure S5b) or Si2p (see Figure S5c) core levels after the oxidative treatment. The survey analysis provided valuable insights into the effects of the oxidative treatment. Remarkably, the presence of residual chlorine exhibited at 201.2 eV (see Figure S5a) and the peak at 531.9 eV in the O1s spectrum (see Figure S5c) ascribed to C = O/Cl-O [60] confirmed the “charge trapping” of the perchlorate counterions introduced during the oxidative swelling process. These counterions became confined within the MPN matrix, even after the discharging of the film. Moreover, a slightly notable alteration in the nitrogen core level was observed at 402.1 eV (see Figure S5d), attributed to the quaternary nitrogen species [70], which suggests that some tetrabutyl ammonium came along the perchlorate anions, becoming trapped inside the MPN film after the process. However, no clear alteration in the chemical state of the PTPTCzSiOH film was observed by the oxidation process.

#### 3.2.2. Surface Morphology of the MPN Films

The surface morphology of the ITO glass electrodes before and after the electrodeposition of polymer films was investigated by scanning electron microscopy (SEM). Figure S6 shows the top-view SEM micrographs of the bare ITO electrodes and the PCBP, PTCB, and PTPTCzSiOH films electrodeposited on the ITO surface. The surface of the ITO was smooth, with little roughness (see Figure S6a) [71]. In general, the morphology of the polymer-modified ITO electrodes was the same as that of the bare electrode, indicating that the film was quite thin. One distinctive feature of these films was the cracking after removal from the polymerization medium, as shown in Figure S6d. On the other hand, in some sectors, the morphology of the film became rougher (see Figure S6b), suggesting polymer agglomeration. In addition, energy dispersive spectroscopy (EDS) analysis was performed to verify the successful electropolymerization of each monomer on the ITO electrode. EDS confirmed the composition of ITO (In 11.77 at%, O 51.03 at%, Si 20.68 at%, Sn 1.42 at%, C 12.52 at%, Ca 2.58 at%)—as the presence of indium, tin, and oxygen was expected for this coating [56,72]—and the silicon and calcium content for the glass substrate [73]. Carbon appeared in the EDS spectrum, presumably due to surface contamination of the electrode during handling for electropolymerization and the previous XPS analysis. Interestingly, an increase in the atomic concentration of carbon was observed for the MPN-modified ITO electrodes, confirming the presence of polymer layers on the ITO surface (21.83 at% for PCBP-ITO, 20.06 at% for PTCB-ITO, 19.51 at% for PTPTCzSiOH -ITO). 

### 3.3. The Electrochemical Detection of Metronidazole

**Figure 6:**
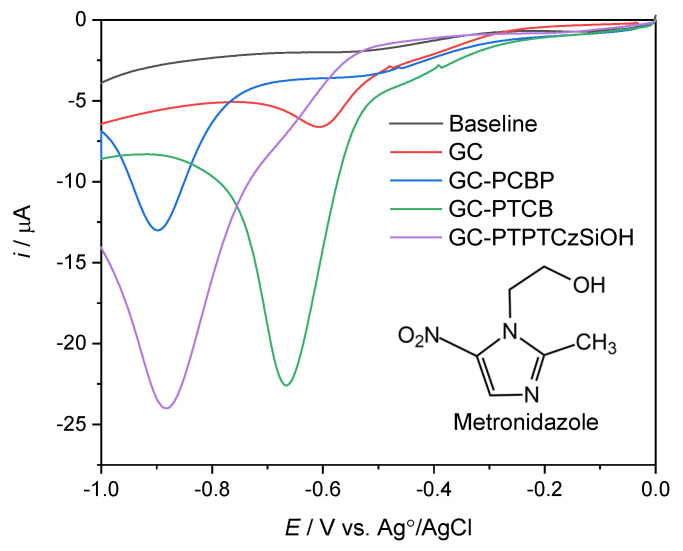
Linear scan voltammograms of bare GCE and GC-PCBP-, GC-PTCB-, and GC-PTPTCzSiOH-modified electrodes for the detection of 50 μM MNZ in 0.1 M PBS (pH 7). The scan rate was 0.1 V/s. Abscissa values in volts vs. Ag/AgCl.

The electrochemical reduction of MNZ was investigated by linear scan voltammograms (LSVs) on GC electrodes coated with PTPTCzSiOH, PCBP, and PTCB deposits. Figure 6 shows LSVs for the reduction of 50 µM MNZ on bare glassy carbon and each polymer-modified electrode. For the bare GCE, a small cathodic wave appeared near −0.6 V vs. Ag/AgCl, which was attributed to a four-electron reduction process of the nitro group (R-NO_2_) to a hydroxylamine group [18,19]. The cathodic peak for the modified GC electrodes shifted at more negative potentials, which is indicative of decreased conductivity, dependent on the structures of each polymer film [33,74]. However, an enhancement in the current response was observed for PTCB-, PCBP-, and PTPTCzSiOH-modified electrodes that was up to 3.6 times higher than the response for the bare GC electrode (see Table S2). This effect was driven by increased interfacial interactions between the high surfaces of electron-rich microporous polymers and the electron-poor structural units of MNZ [33,34,74].

### 3.4. Computational Approach

#### 3.4.1. Ground State Geometric Optimization and Frontier Molecular Orbitals (FMOs)

**Figure 7:**
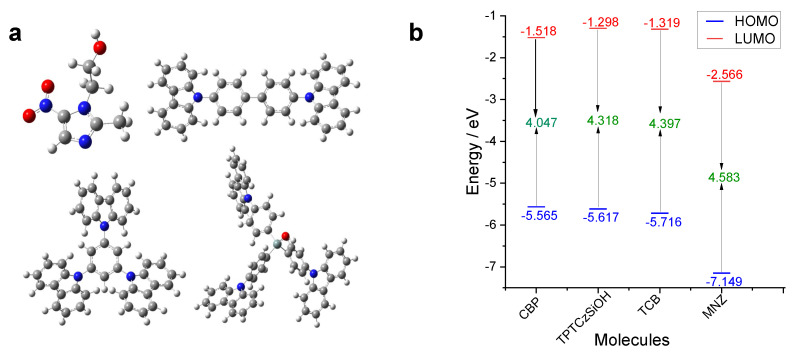
(**a**) Optimized structures of analyte and monomer molecules acquired applying DFT/B3LYP/def2-TZVP level of theory. Color codes: C (grey), H (white), N (blue), O (red). (**b**) HOMO and LUMO energies of the investigated molecules: CBP, TPTCzSiOH, TCB, and MNZ. The band gap energy is also represented.

The molecular structures of the MNZ analyte and CBP, TCB, and TPTCzSiOH monomers were optimized using the DFT/B3LYP/def2-TZVP level of theory with the ORCA 5.0 package, as shown in Figure 7a. In this way, structural parameters such as bond length and dihedral angle were determined from the theoretical spatial arrangements of the atoms in the optimized molecules. The highest occupied molecular orbitals (HOMOs) and lowest unoccupied molecular orbitals (LUMOs) for monomers and analyte molecules were determined using the DFT/B3LYP method with the def2-TZVP basis set. The HOMO and LUMO energy levels, including the bandgap energy (E_g_) between them, are illustrated in Figure 7b. The HOMO energy of all three monomers was higher than that of MNZ, indicating that monomers were the main electron donors. On the contrary, the LUMO energy of MNZ was lower than that of the monomers, demonstrating that MNZ mainly acted as an electron acceptor [75]. The E_g_ for the investigated monomers was predicted to be in the following order: TCB > TPTCzSiOH > CBP. The increase in the π-conjugation of molecules has been associated with a drop in bandgap energy [76]. This indicates that CBP possessed the highest π-conjugation, followed by the TPTCzSiOH and TCB monomers, allowing more electron transfer from CBP to MNZ in the electrochemical reduction process. All three monomers had a small and close band gap value ranging from 4.047 eV to 4.397 eV; therefore, upon polymerization, it would be expected that they give rise to (semi)conducting films, which is in agreement with the experimental results.

Electron-donating and electron-withdrawing groups directly influence frontier molecular orbitals (FMOs), mainly by increasing the HOMO and reducing the LUMO energies, respectively [77]. HOMO energy is associated with a molecule’s ability to donate electrons [78]; the higher the value of E_HOMO_, the lower the onset oxidation potential value ($E_{ox}^{onset}$) [79]. In this context, the oxidation potential for the monomers should have had the following tendency: TCB > TPTCzSiOH > CBP. However, the experimental $E_{ox}^{onset}$, taken from the first anodic scan in the polymerization of the three monomers, show contrary behavior for CBP and TPTCzSiOH; the value of onset oxidation potential of TPTCzSiOH was found to be lower than that of CBP (see Table S3). Theoretically, the HOMO energy for the monomers was predicted to be −5.565 eV, −5.617 eV, and −5.716 eV for CBP, TPTCzSiOH, and TCB, respectively. As expected, TCB had the lowest HOMO energy value (experimental E_HOMO_ = −6.104 eV); however, for CBP and TPTCzSiOH, the theoretical values did not completely align with the experimental ones, as in the case of $E_{ox}^{onset}$. The difference between the theoretical and experimental trends of the oxidation potential may be associated with the experimental conditions (solvent, pH, temperature, and electrolyte) used for electropolymerization. These factors, as well as the molecular structure, substituting effect, and electron-donating or withdrawing groups, may interact with each other, making the prediction of oxidative potential more complex [75,80]. Although the trends for the experimental and theoretical HOMO were not completely aligned, the values were very close to each other (see Table S3), considering that computations were performed in the gas phase. This gave at least a raw idea of the potential needed to initiate the polymerization.

The FMO contours for the CBP, TCB, and TPTCzSiOH monomers are demonstrated in Figure S7. The HOMO orbital density of the CBP molecule was symmetrically delocalized along the chain, with higher orbital densities over the electron donor (carbazole) subunit. TCB also showed orbital delocalization in the whole molecule; however, one of the carbazole subunits had a higher HOMO orbital density. In contrast, the HOMO orbital density of TPTCzSiOH was not distributed but localized in one of the three 9-phenylcarbazole subunits. Although the low HOMO delocalization in the last monomer may be explained by the low molecular symmetry, it is worth noting that the energies of HOMO, HOMO-1, and HOMO-2 (which were distributed in the three carbazole units) were close together, and even those of HOMO and HOMO-1 were within Orca’s margin of error. A common feature of all three monomers was the high HOMO orbital density in the carbazole subunit, where the linking sites were located [34]. This made them more reactive and facilitated bond formation between monomers at those positions. An analysis of the FMO of MNZ indicated that the HOMO was mainly distributed in the heterocyclic ring region. On the contrary, the LUMO orbital density was strongly distributed in the NO_2_ group. Therefore, the nitro group had a high tendency to accept electrons and reduce to a hydroxylamine group, giving rise to the cathodic response in electrochemical detection [78,79]. 

#### 3.4.2. Molecular Electrostatic Potential (MEP)

**Figure 8:**
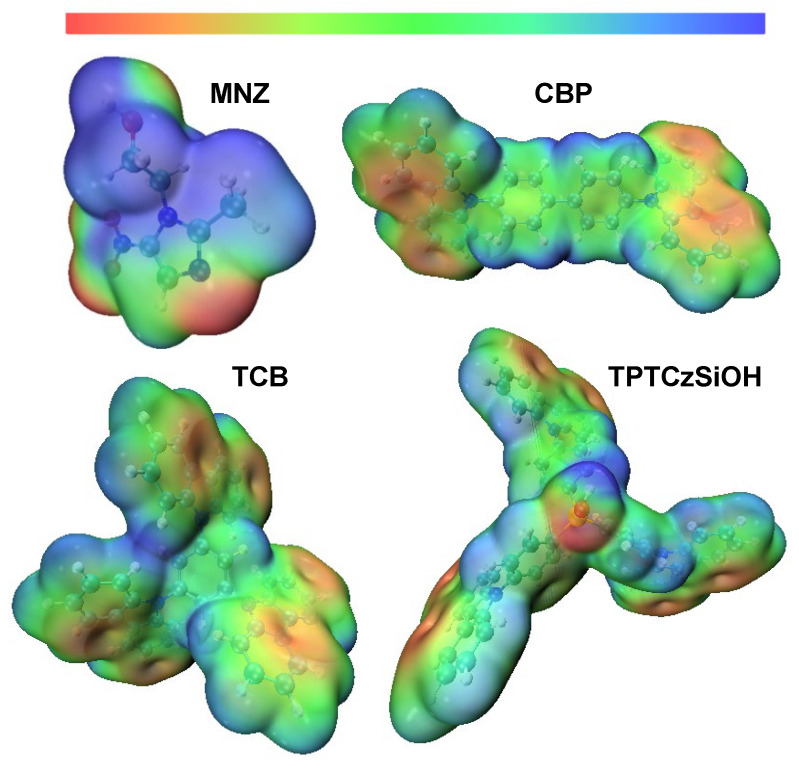
Molecular electrostatic potential surface maps for analyte and monomer molecules in the ground state acquired applying the DFT/B3LYP level of theory.

MEP was determined to investigate the chemical properties of analyte and monomer molecules. MEP is a representation of the electron potential on electron density surfaces that displays the charge distributions of molecules and provides information about structure, reactivity, and electrostatic properties [76]. Figure 8 shows the 3D MEP surfaces of the investigated donor and acceptor molecules in the ground state. MEP surfaces are presented with different color codes that determine the electron density of regions. The blue color denotes an electron-poor region with positive electrostatic potential while the red color corresponds to an electron-dense region with negative electrostatic potential. As depicted in Figure 8, monomers possessed electron-rich areas located at the π-bonds, mainly in carbazole subunits, while analyte was characterized by a wide electron-deficient area. These features in conjunction with the high surface areas account for the enhancement of the current response in the electrochemical sensing of MNZ [31,34,74]. Based on the BET surface areas of 104 m^2^g^−1^ for PCBP, 165 m^2^g^−1^ for PTPTCzSiOH, and 748 m^2^g^−1^ for PTCB, one would have expected the PTCB-modified electrode to give a higher detection signal. However, the experimental data show a higher detection signal for GCE modified by PTPTCzSiOH, followed by that modified by PTCB and PCBP; this may be explained by the interaction sites that the monomers possessed. CBP had two electron-rich interaction sites localized on the carbazole units. TCB exhibited three interaction sites localized on the carbazole units. The TPTCzSiOH monomer not only showed three interaction sites of the carbazole units but also possessed a fourth electron-rich interaction site located in the oxygen from the silanol group, which can be related to the slight increase in current response in comparison to TCB. Moreover, the silanol group enhanced the hydrophilicity of the PTPTCzSiOH film, increasing its compatibility with aqueous media and boosting the current response.

## 4. Conclusions

Thin films of poly-4,4′-Di(carbazol-9-yl)-1,1′-biphenyl, poly-1,3,5-tri(carbazol-9-yl)benzene, and poly-tris(4-(carbazol-9-yl)phenyl)silanol were electrochemically generated on glassy carbon and ITO electrodes. SEM, EDS, and XPS analyses were conducted for all the polymer films, confirming the successful electropolymerization of the CBP, TCB, and TPTCzSiOH monomers. In addition, it was found that the anodic switching potential for the electropolymerization of monomers could be changed within a broad range, without changing their electrochemical activity. CBP presented the widest range, from 0.9 to 1.6 V, while the lowest range was for TCB, from 0.95 to 1.3 V. The electrochemical stability and reversibility of the polymeric films were relatively low. A decrease in the anodic and cathodic charges was observed during all thirty cycles used for stability and reversibility analyses, which was more pronounced for the PTCB-modified electrode. This behavior was attributed to the non-purged conditions, the loss of soluble electroactive material from the electrode surface, and, mainly, the charge-trapping phenomenon. However, the scan rate analysis showed that the films adhered well to the electrode surface. 

MPN-modified GCEs proved to be promising candidates as electrochemical sensors for MNZ. The microporous polymer films conferred a large electroactive surface area to the GCE, increasing the detection signal of the nitrocompound compared to the bare electrode. GC-PTPTCzSiOH was the best candidate in terms of cathodic current response, but, energetically, GC-PTCB offered better results since the overpotential required for MNZ reduction was the lowest of the three modified electrodes. 

This DFT-based computational study provides valuable information for the prediction and understanding of polymer film properties. The order of the potential required for monomer oxidation was partially predicted. Through the frontier orbitals, it was confirmed that the nitro acceptor group of MNZ is associated with the electron transfer process in electrochemical reduction. MEP surfaces provided a graphical picture of the electron-rich regions of the monomers and the electron-poor sites of MNZ responsible for increased signal detection. Additionally, a possible explanation for the higher signal obtained by GC-PTPTCzSiOH is the extra interaction site delivered by the silanol group. In terms of implementation as sensors, the low stability of the polymer-modified electrodes represents a drawback for reusability; however, further studies should be carried out under different synthesis conditions, e.g., copolymerization with appropriate co-monomers and nanocomposites with nanocarbons and metal nanoparticles.

## Data Availability

Data are available from the authors upon request.

## Supplementary Materials

The following supporting information can be downloaded at: https://www.mdpi.com/article/10.3390/nano14020180/s1, Figure S1: FT-IR spectrum of purified metronidazole. The 1263.57 cm^−1^ and 1185.29 cm^−1^ peaks correspond to the bonds –C=C– and –C=N– of the imidazole cycle. The broad absorption band at 3211.10 cm^−1^ and the peak at 3099.28 cm^−1^ indicate the presence of hydroxyl group. Absorption bands at 1533.8 cm^−1^ and 1366.07 cm^−1^ correspond to the stretching vibrations of the NO_2_ group; Figure S2: NMR spectrum for purified metronidazole. 1H NMR (60 MHz, CHLOROFORM-d) δ ppm 2.51 (s, 3 H) 3.74–4.19 (m, 2 H) 4.23–4.68 (m, 2 H) 7.90 (s, 1 H). No hydroxyl proton signal was identified; Figure S3: Electrochemical stability of (a) PCBP, (b) PTCB, and (c) PTPTCzSiOH films on GCE obtained in a free monomer solution, ACN/TBAP 0.1 M. The applied potential range was 0–1.0 V with a sweeping rate of 0.1 V/s. The variation of the anodic and cathodic charge (Qa and Qc, left axis) and the Qc/Qa (right axis, blue curve) during the 30 cycles for (d) PCBP, (e) PTCB, and (f) PTPTCzSiOH films on GCE are also shown; Table S1: Electrochemical stability and reversibility of polymer films in ACN/TBAP 0.1M represented as the percentage of cathodic current loss between the second and thirtieth cycle (%Qc), and cathodic to anodic charge ratio at the thirtieth cycle (Qc/Qa), respectively; Figure S4: (a) Electropolymerization of TPTCzSiOH film on ITO scanning from 0 V vs. Ag/AgNO3 to 1.1 V for 20 cycles at 0.1 Vs^−1^ in a 0.1 M TBAP/ACN solution with 0.1 mM monomer concentration. (b) Chronoamperograms for thin ITO-PTPTCzSiOH films with an oxidative potential applied of 1 V for 30 min followed by a dedoping potential of 0 V for 5 min; Figure S5: XPS spectra of the (a) survey scan, and high-resolution spectra of (b) C1s, (c) N1s, (d) Si2p, and (e) O1s of ITO- PTPTCzSiOH electrode after the oxidative treatment described in Figure S4; Figure S6: The top-view SEM topographies of (a) bare ITO, (b) PCBP-ITO, (c) PTCB-ITO, and (d) PTPTCzSiOH-ITO electrodes; Table S2: Current response for the cathodic reduction peak in the electrochemical detection of 50 µM MNZ. The current ratios of the peak between MPN-modified and non-modified GC electrodes are also listed; Table S3. Experimental and theoretical values of the onset oxidation potential (Eonset) and the energy of HOMO orbital (EHOMO); Figure S7: The contour representation of frontier molecular orbitals computed by DFT/B3LYP method for investigated molecules. (See Refs. [81,82,83]).
