# Supplementary material for: Microporous Polymer-Modified Glassy Carbon Electrodes for the Electrochemical Detection of Metronidazole: Experimental and Theoretical Insights
Source: Nanomaterials (Basel). 2024 Jan 12;14(2):180. doi: 10.3390/nano14020180 (PMC10819510; doi:10.3390/nano14020180)
Supplement: Supplementary file 1 [file nanomaterials-14-00180-s001.zip › nanomaterials-2758360-supplementary.pdf]

SUPPLEMENTARY MATERIALS

Microporous Polymer Modified Glassy Carbon Electrodes for Electrochemical Detection of Metronidazole: Experimental and Theoretical Insights

Héctor Quiroz-Arturo [1], Carlos Reinoso [2], Ullrich Scherf [3,*], and Alex Palma-Cando [1,*]

[1] Grupo de Investigación Aplicada en Materiales y Procesos (GIAMP), School of Chemical Sciences and Engineering, Yachay Tech University, Hda. San José s/n y Proyecto Yachay, 100115 Urcuqui, Ecuador

[2] School of Physical Sciences and Nanotechnology, Yachay Tech University, Hda. San José s/n y Proyecto Yachay, 100115 Urcuqui, Ecuador

[3] Department of Chemistry, Macromolecular Chemistry and Wuppertal Center for Smart Materials @ Systems (CM@S), Bergische Universität Wuppertal, Gaußstr. 20, 42119 Wuppertal, Germany

* Correspondence: scherf@uni-wuppertal.de (U. S.); apalma@yachaytech.edu.ec (A. P.-C.)

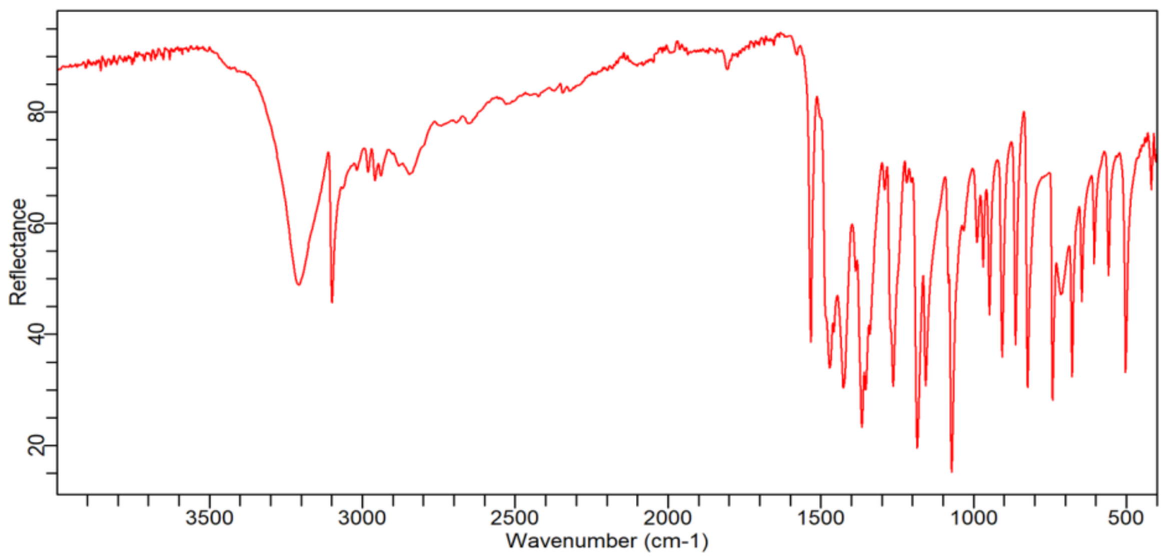

Figure S1. FT-IR spectrum of purified metronidazole. The 1263.57 cm^{-1} and 1185.29 cm^{-1} peaks correspond to the bonds –C=C– and –C=N– of the imidazole cycle. The broad absorption band at 3211.10 cm^{-1} and the peak at 3099.28 cm^{-1} indicate the presence of hydroxyl group. Absorption bands at 1533.8 cm^{-1} and 1366.07 cm^{-1} correspond to the stretching vibrations of the NO_2 group [S1,S2].

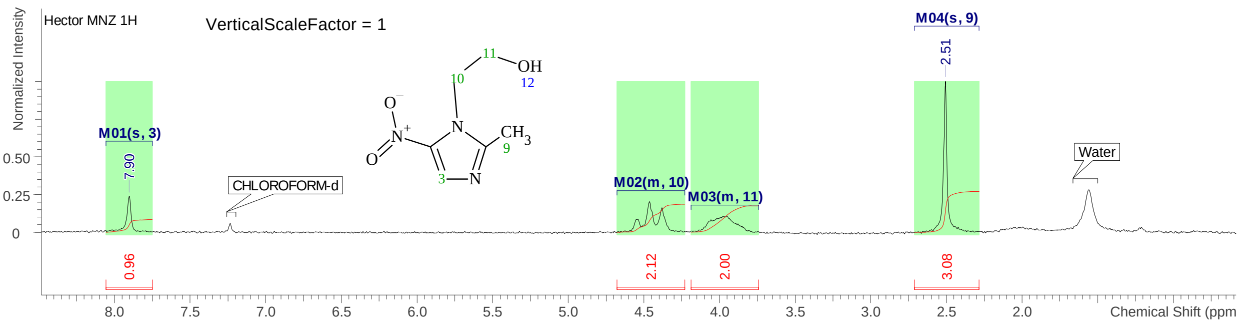

Figure S2. NMR spectrum for purified metronidazole. ^{1}H NMR (60 MHz, CHLOROFORM-d) δ ppm 2.51 (s, 3 H) 3.74 - 4.19 (m, 2 H) 4.23 - 4.68 (m, 2 H) 7.90 (s, 1 H). No hydroxyl proton signal was identified [S3].

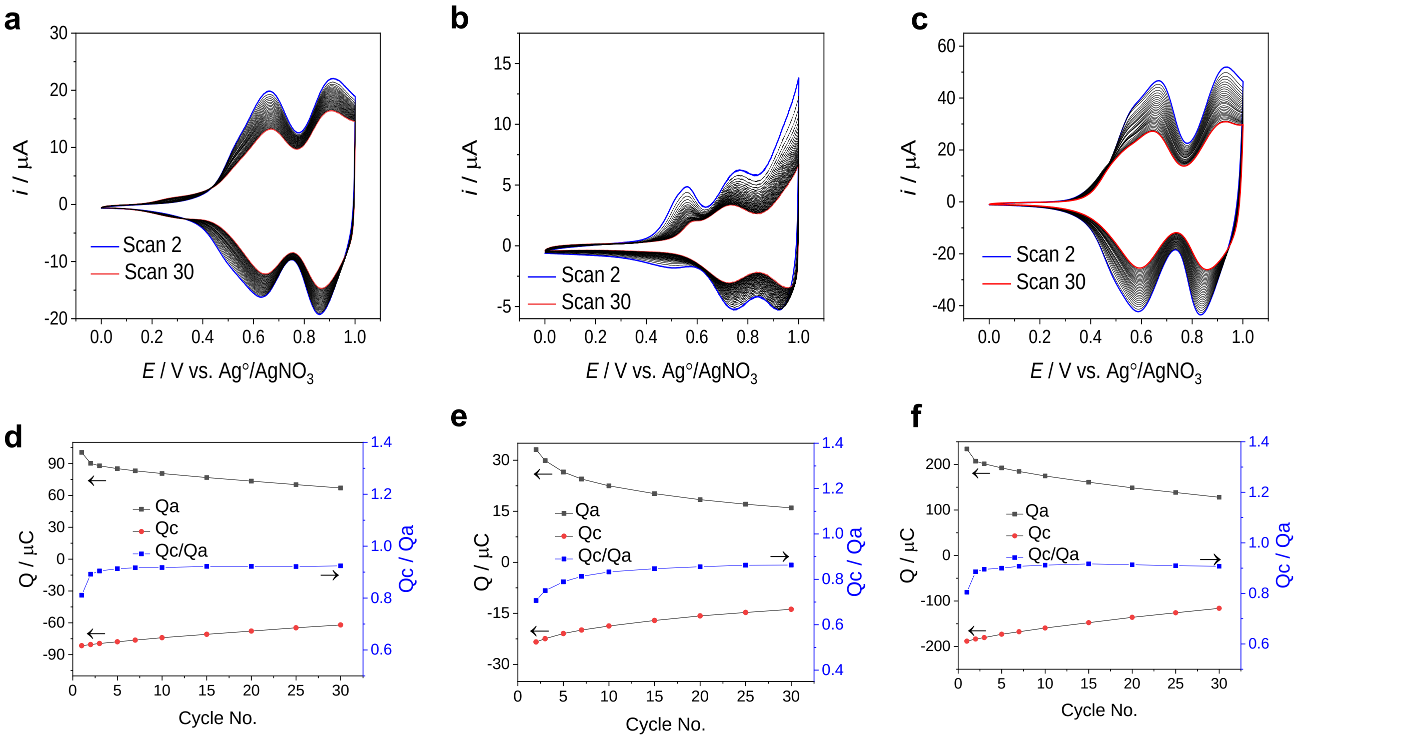

Figure S3. Electrochemical stability of (a) PCBP, (b) PTCB, and (c) PTPTCzSiOH films on GCE obtained in a free monomer solution, ACN/TBAP 0.1 M. The applied potential range was 0 – 1.0 V with a sweeping rate of 0.1 V/s. The variation of the anodic and cathodic charge (Qa and Qc, left axis) and the Qc/Qa (right axis, blue curve) during the 30 cycles for (d) PCBP, (e) PTCB, and (f) PTPTCzSiOH films on GCE are also shown.

Table S1. Electrochemical stability and reversibility of polymer films in ACN/TBAP 0.1M represented as the percentage of cathodic current loss between the second and thirtieth cycle (%Qc), and cathodic to anodic charge ratio at the thirtieth cycle (Qc/Qa), respectively.

Polymer	% Qc	Qc/Qa
PCBP	23.0	0.92
PTCB	40.9	0.86
PTPTCzSiOH	36.7	0.91

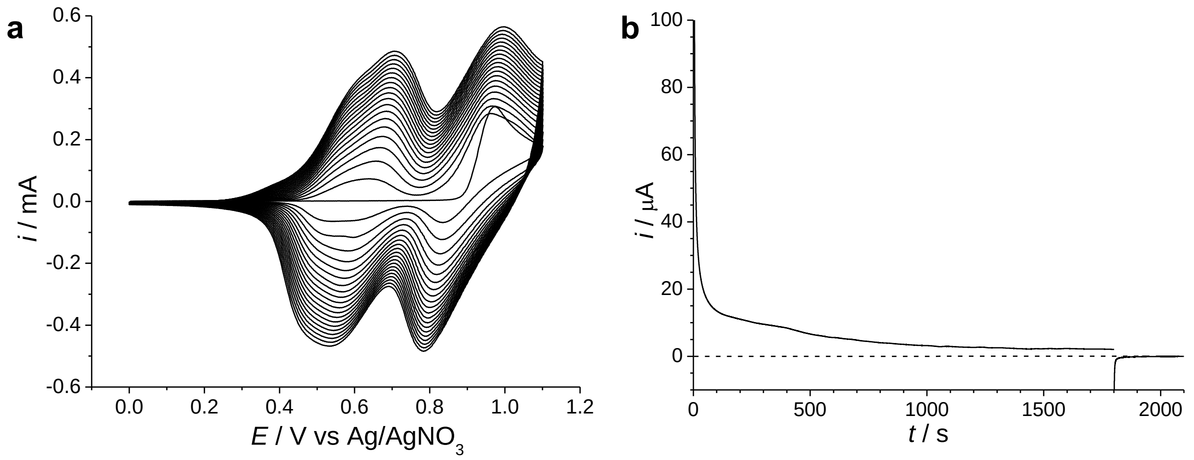

Figure S4. (a) Electropolymerization of TPTCzSiOH film on ITO scanning from 0 V vs. Ag/AgNO3 to 1.1 V for 20 cycles at 0.1 Vs-1 in a 0.1 M TBAP/ACN solution with 0.1 mM monomer concentration. (b) Chronoamperograms for thin ITO-PTPTCzSiOH films with an oxidative potential applied of 1 V for 30 min followed by a dedoping potential of 0 V for 5 min.

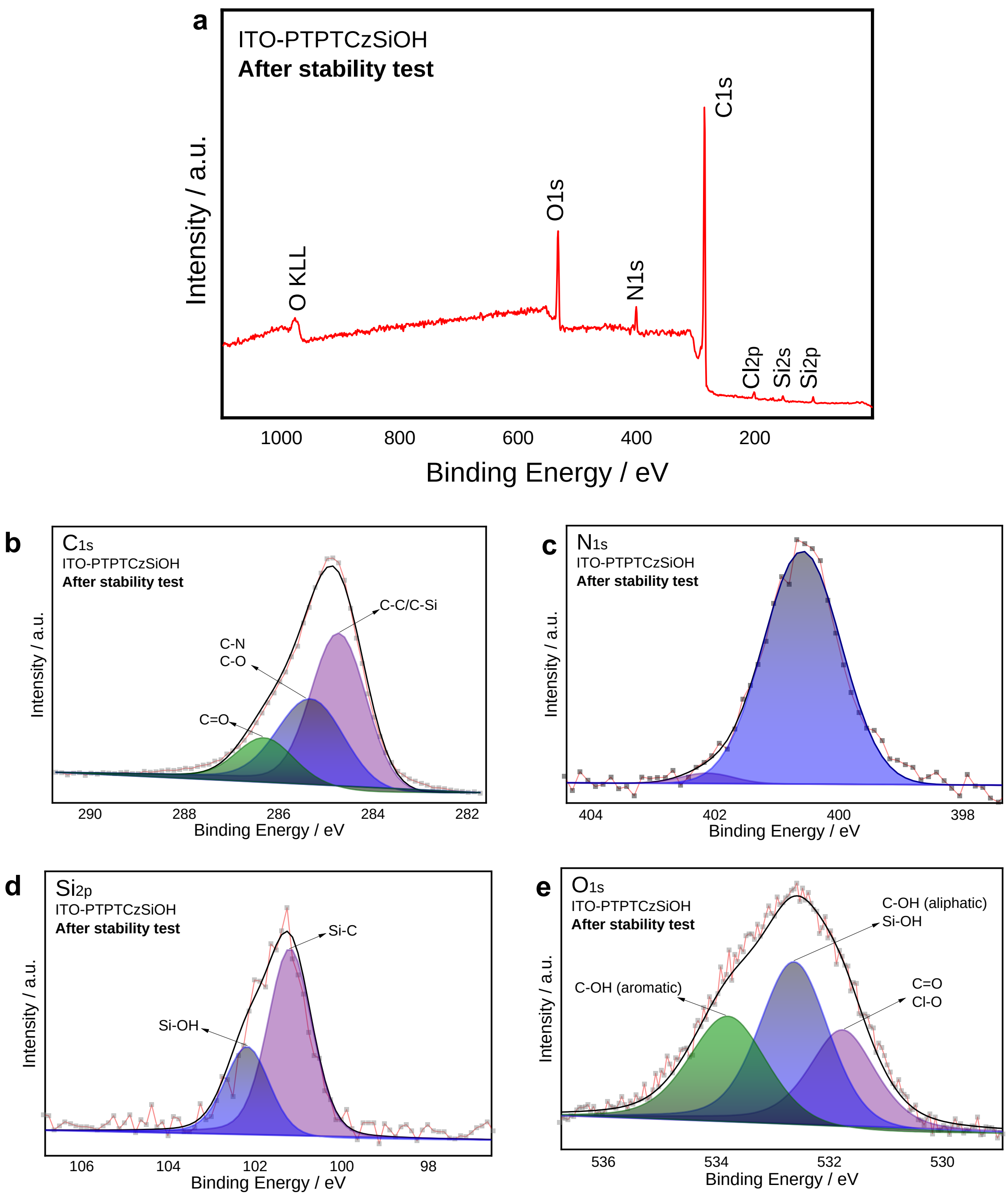

Figure S5. XPS spectra of the (a) survey scan, and high-resolution spectra of (b) C1s, (c) N1s, (d) Si2p, and (e) O1s of ITO- PTPTCzSiOH electrode after the oxidative treatment described in Figure S4.

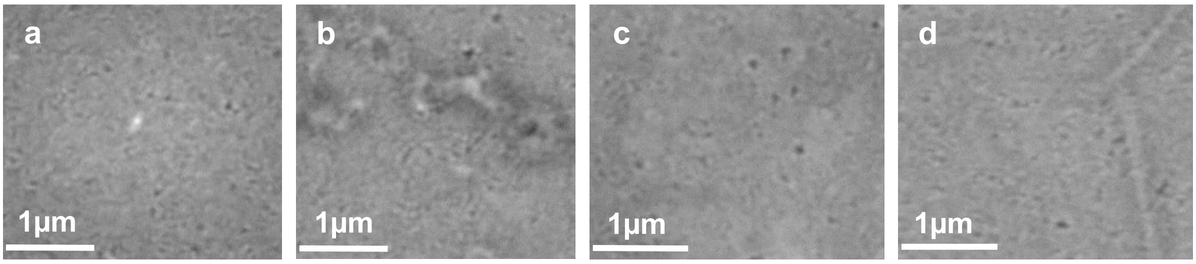

Figure S6. The top-view SEM topographies of (a) bare ITO, (b) PCBP-ITO, (c) PTCB-ITO, and (d) PTPTCzSiOH-ITO electrodes.

Table S2. Current response for the cathodic reduction peak in the electrochemical detection of 50 µM MNZ. The current ratios of the peak between MPN-modified and non-modified GC electrodes are also listed.

Electrode	E_p (V)	i_p (µA)	$i_p / i_{p\ GC}$
**GC**	-0.60	-6.60	1.0
**GC-PCBP**	-0.89	-13.0	1.9
**GC-PTCB**	-0.66	-22.6	3.4
**GC-PTPTCzSiOH**	-0.88	-23.9	3.6

Table S3. Experimental and theoretical values of the onset oxidation potential (E_{onset}) and the energy of HOMO orbital (E_{HOMO}).

Monomer	E_{onset} (V)	E_{HOMO} (eV) experimental	E_{HOMO} (eV) theoretical
CBP	0.86	-5.96	-5.57
TCB	1.00	-6.10	-5.72
TPTCzSiOH	0.85	-5.95	-5.62

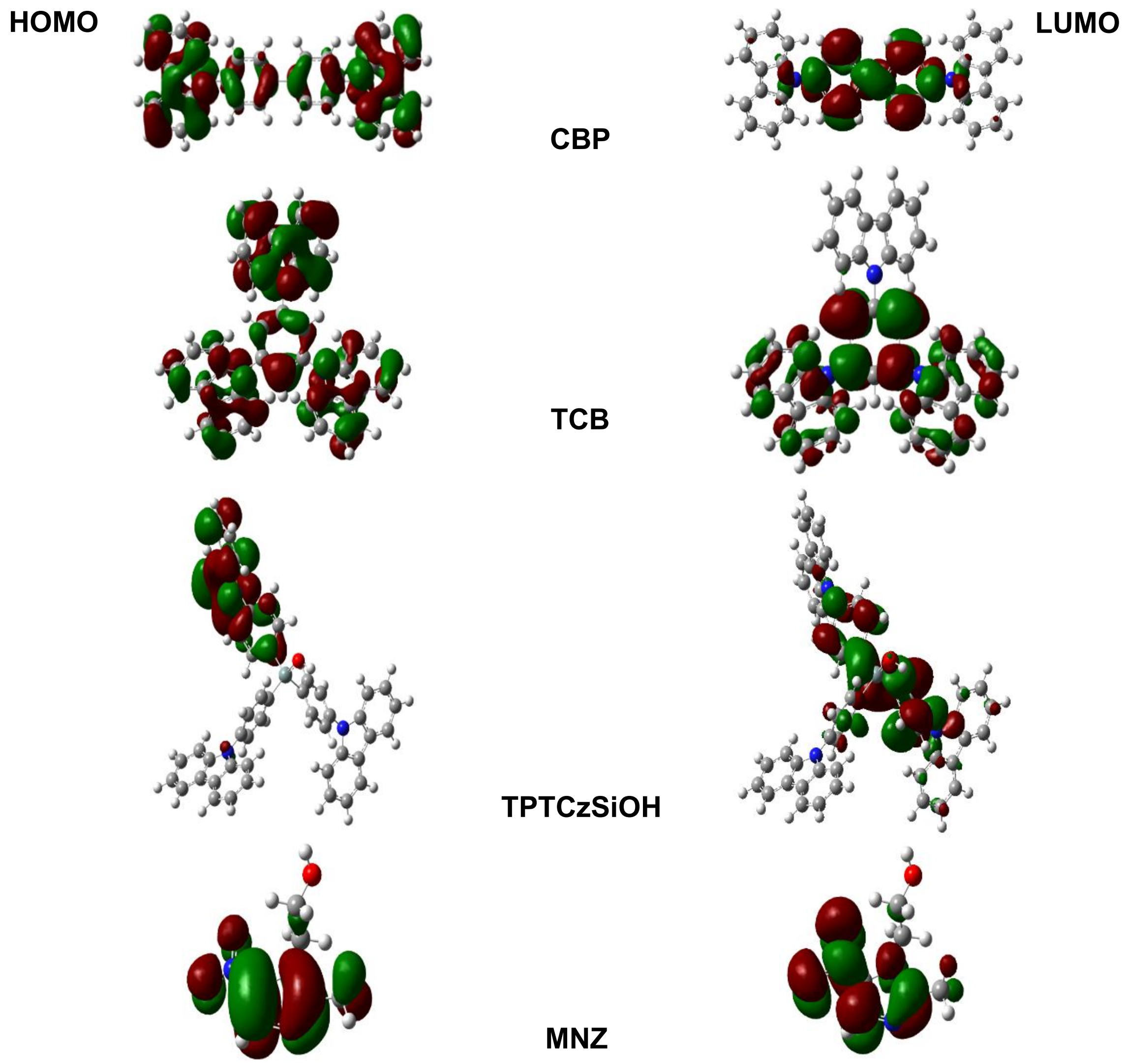

Figure S7. The contour representation of frontier molecular orbitals computed by DFT/B3LYP method for investigated molecules.

References

S1. Myhal, A. V.; Golovchenko, O.S.; Krutskikh, T. V.; Gubar, S.M.; Georgiyants, V.A. IR-Spectroscopy Research into the Structure of Products of Interaction between Metronidazole and Metal Salts. *Der Pharma Chemica* **2016**, *8*, 148–154.

S2. Szente, V.; Baska, F.; Zelkó, R.; Süvegh, K. Prediction of the Drug Release Stability of Different Polymeric Matrix Tablets Containing Metronidazole. *Journal of Pharmaceutical and Biomedical Analysis* **2011**, *54*, 730–734, doi:10.1016/j.jpba.2010.11.005.

S3. Kalinowska-Lis, U.; Felczak, A.; Checińska, L.; Zawadzka, K.; Patyna, E.; Lisowska, K.; Ochocki, J. Synthesis, Characterization and Antimicrobial Activity of Water-Soluble Silver(I) Complexes of Metronidazole Drug and Selected Counter-Ions. *Dalton Transactions* **2015**, *44*, 8178–8189, doi:10.1039/c5dt00403a.
